# Analysis of clinical characteristics of different types of lung function impaiement in TDL patients

**DOI:** 10.1186/s12890-024-03115-5

**Published:** 2024-06-24

**Authors:** Jing Zhao, Xiaoman Cao, YunSong Li, Yang Li, Teng Ma, Fangchao Liu, Hongyun Ruan

**Affiliations:** 1 Department of anesthesia, Beijing Chest Hospital, Beijing Tuberculosis and Thoracic Tumor Research Institute, Capital Medical University, Beijing, P. R. China; 2 Department of Thoracic Surgery, Beijing Chest Hospital, Beijing Tuberculosis and Thoracic Tumor Research Institute, Capital Medical University, Beijing, P. R. China; 3 Department of General, Changchun Infectious Disease Hospital, Changchun city, Jilin P. R. China; 4 Department of Science and Technology, Beijing Chest Hospital, Beijing Tuberculosis and Thoracic Tumor Research Institute, Capital Medical University, Beijing, P. R. China; 5 Department of Cellular and Molecular Biology, Beijing Chest Hospital, Beijing Tuberculosis and Thoracic Tumor Research Institute, Capital Medical University, Beijing, P. R. China; 6 No 9, Bei guan Street, Tong Zhou District, Beijing, 101149 P. R. China

**Keywords:** Tuberculosis-destroyed lung, Pulmonary function test, Restrictive spirometric pattern, Obstructive spirometric pattern

## Abstract

**Aim:**

The clinical characteristics associated with pulmonary function decline in patients with Tuberculosis-destroyed lung (TDL) remain uncertain. We categorize them based on the pattern of pulmonary function impairment, distinguishing between restrictive spirometric pattern (RSP) and obstructive spirometric pattern (OSP). We aim to compare the severity of these patterns with the clinical characteristics of TDL patients and analyze their correlation.

**Method:**

We conducted a retrospective analysis on the clinical data of TDL patients who underwent consecutive pulmonary function tests (PFT) from November 2002 to February 2023. We used the lower limit formula for normal values based on the 2012 Global Lung Function Initiative. We compared the clinical characteristics of RSP patients with those of OSP patients. The characteristics of RSP patients were analyzed using the tertiles of forced vital capacity percentage predicted (FVC% pred) decline based on PFT measurements, and the characteristics of OSP patients were analyzed using the tertiles of forced expiratory volume in 1 s percentage predicted (FEV_1_% pred) decline.

**Result:**

Among the RSP patients, those in the Tertile1 group (with lower FVC% pred) were more likely to have a higher of body mass index (BMI), spinal deformities, and C-reactive protein (CRP) compared to the other two groups (*P for trend* < 0.001, 0.027, and 0.013, respectively). Among OSP patients, those in the Tertile1 group (with lower FEV_1_% pred) showed an increasing trend in cough symptoms and contralateral lung infection compared to the Tertile 2–3 group (*P for trend* 0.036 and 0.009, respectively).

**Conclusion:**

For TDL patients, we observed that Patients with high BMI, a higher proportion of spinal scoliosis, and abnormal elevation of CRP levels were more likely to have reduced FVC. Patients with decreased FEV_1_% pred have more frequent cough symptoms and a higher proportion of lung infections on the affected side.

## Introduction

Tuberculosis destroyed lung (TDL) is currently known as one of the most severe complications of pulmonary tuberculosis [1, 2]. Despite the continuous release of new diagnostic and treatment guidelines by the World Health Organization for tuberculosis, there are still various factors such as uncontrollable multidrug-resistant pulmonary tuberculosis and severe drug reactions [3–6]. As a result, 40.3–66.7% of pulmonary tuberculosis patients may experience extensive lung structural changes, and approximately 1.3% of pulmonary tuberculosis patients progress to TDL [7, 8]. TDL patients exhibit significant impairment of lung function and irreversible necrosis, which is common in the left lung, characterized by cystic bronchiectasis, the coexistence of fibrotic cavities and a caseous focus or reduced ipsilateral lung volume [9–11]. About two-thirds to three-quarters of TDL patients experience chest tightness and difficulty breathing, with some patients being unable to perform activities of daily living and requiring long-term home oxygen therapy [7, 12].

TDL lung dysfunction can manifest as either restrictive [RSP] or obstructive [OSP] form [13]. RSP type lung disease is classified based on forced vital capacity as a percentage of predicted value (FVC% pred), while OSP type lung disease is categorized based on forced expiratory volume in 1 s as a percentage of predicted value (FEV_1_% pred), providing an objective assessment of the patients’ clinical characteristics [14–16].

Pulmonary function testing (PFT) is a commonly used diagnostic method for measuring and monitoring chronic lung diseases in clinical practice [17]. Based on the characteristics of lung function, chronic lung diseases can be classified into restrictive lung diseases (such as sarcoidosis and interstitial lung disease) and obstructive lung diseases (such as chronic obstructive pulmonary disease and bronchiectasis) [18]. However, unlike other chronic lung diseases, the clinical significance of PFT results in patients with TDL (to be defined) is still unclear. Therefore, this study retrospectively analyzed the clinical data of patients who were hospitalized and underwent PFT for TDL from November 2002 to February 2023 in our hospital. Through the correlation between lung function impairment and clinical characteristics, we aim to better observe the disease progression in patients with TDL.

## Materials and methods

### Study subjects

**Fig. 1:**
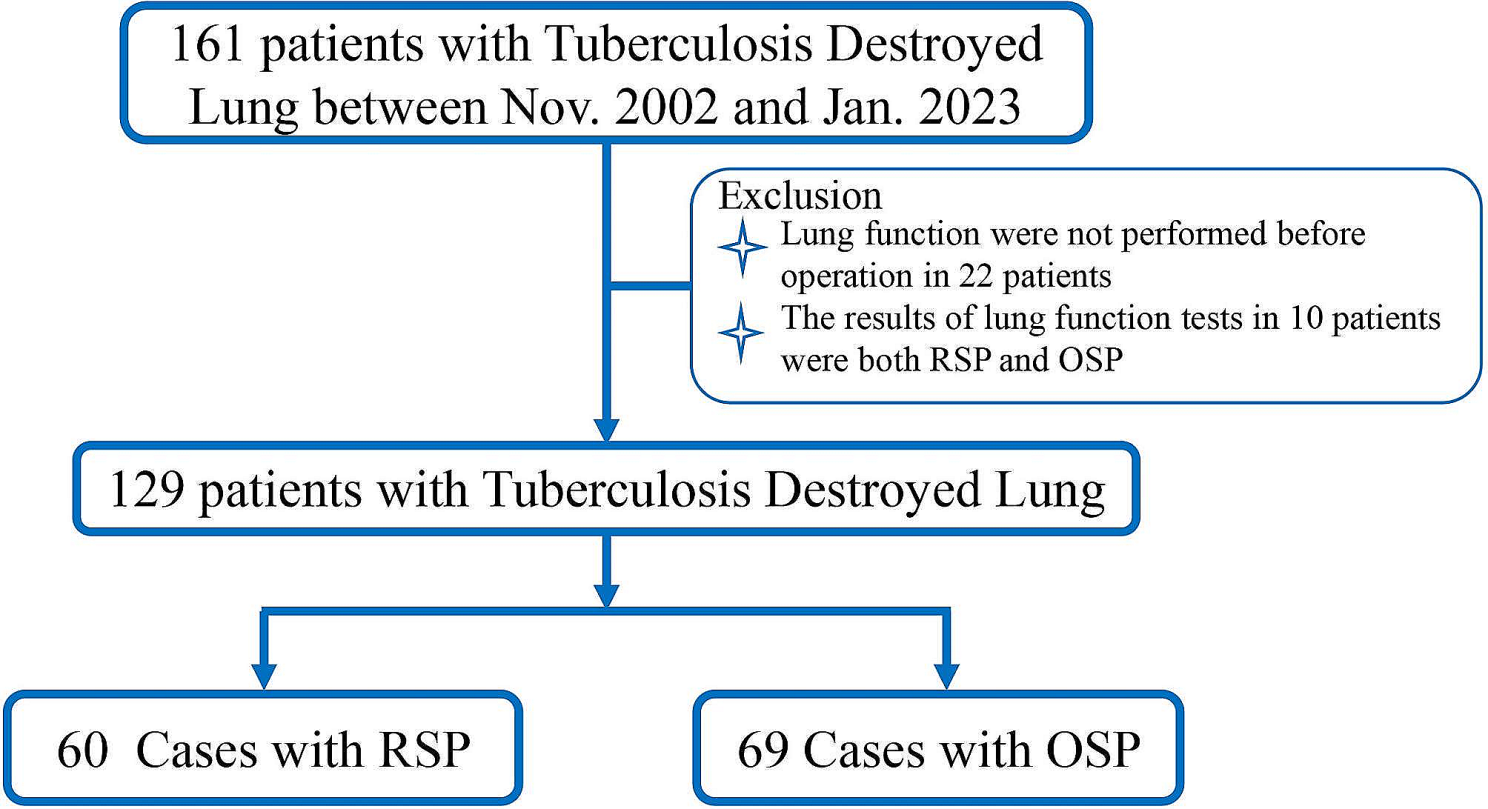
Flowchart of lung function impaiement in TDL patients

Through the hospital’s electronic medical record system, we consecutively included TDL inpatients who underwent preoperative PFT from November 2002 to February 2023 and collected relevant clinical data at Beijing Chest Hospital, Capital Medical University. We screened 161 TDL patients who underwent selective operation, either lobectomy or pneumonectomy. Exclusion criteria: Lung function were not performed before operation in 22 patients (PFT, *n* = 22) and the results of lung function tests in 10 patients were both RSP and OSP (*n* = 10), a total of 129 patients were included in the study, Fig. 1.

The study was designed in accordance with the Helsinki Declaration. The written informed consent was waived by the Beijing Chest Hospital of Capital Medical University of the committee/IRB, (Ethics number: Clinical Research 2018 (43)).

**Fig. 2:**
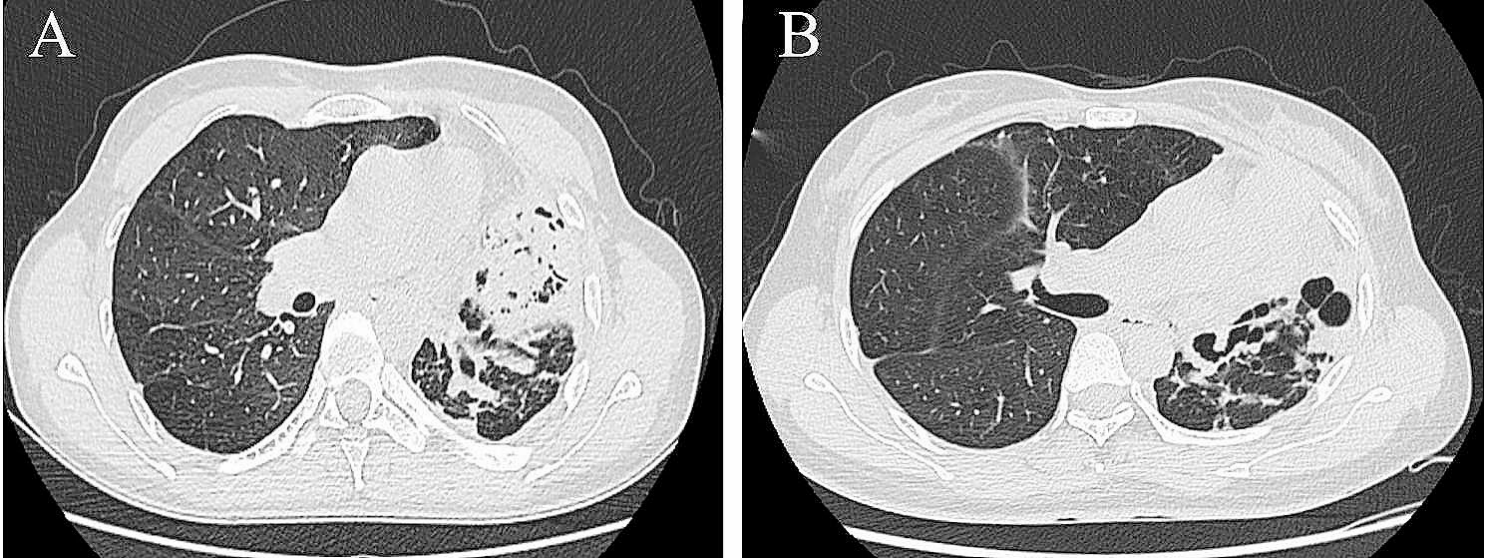
CT of the chest of two of the TDL patients in this study. **A**: A 43-year-old female with TB history for 120 months. Non-enhanced CT shows A solid, air-containing shadow is seen in the upper lobe of the left lung, with a linear air-containing shadow and what appears to be a hollow contour. **B**: A 30-year-old female with TB history for 84 months. Non-enhanced CT shows solid changes with cavitation in the upper lobe of the left lung, Upper lobe of the left lung, striations in the lower lobe, lung destruction, bronchiectasis, etc.

TDL diagnosis: Clinical decisions require the integration of clinical, radiological, and microbiological parameters. TDL diagnosis is based on imaging findings, characterized by cystic bronchiectasis and reduced ipsilateral lung volume. [11] Some patients may also present with fused giant cavities, and rarely, there may be large cavities that span interlobar fissures [8, 19]. TDL is typically associated with irreversible lung function loss resulting from chronic pulmonary infections. exhibit postoperatively determined pathomorphologic disease features consistent with tuberculosis (TB), Fig. 2.

Data collection: General clinical information of patients including gender, age, body mass index (BMI), smoking, alcohol consumption, history of tuberculosis, and major comorbidities (hypertension, coronary heart disease, and diabetes). Chronic respiratory symptoms include fever, cough, and intermittent hemoptysis. Dyspnea index assessment (based on the modified Medical Research Council (mMRC) grading scale). CT imaging findings include presence of cavities, existence of scoliosis, and contralateral lung infection. Laboratory tests include C-reactive protein (CRP), hemoglobin, and albumin.

Pulmonary function testing: The pulmonary function testing instruments used are the Master Screen-IOS and Master Screen-PFT, produced by Jaeger, Germany. These devices employ the single breath technique for measuring lung diffusion. The PFT procedures are performed by trained technicians who hold a valid certificate for pulmonary function training. The tests are conducted on patients whose condition permits pulmonary function testing. The absolute values of FVC and FEV_1_ are measured and compared to the lower limit of normal (LLN) values based on the Global Lung Function Initiative’s reference equations from 2012 [20]. The percentage of measured FVC and FEV_1_ values to predicted values is calculated, as well as the LLN value (which is below the fifth percentile of healthy non-smoking subjects, corresponding to a z-score of -1.64). The definition of a normal spirometry pattern (NSP) is when the post-bronchodilator FEV_1_/FVC ratio is greater than or equal to LLN and FVC is greater than or equal to LLN. The definition of RSP is when the post-bronchodilator FEV_1_/FVC ratio is greater than or equal to LLN, but FVC is less than LLN. The definition of OSP is when the post-bronchodilator FEV_1_/FVC ratio is less than LLN and FEV_1_ is less than LLN. In statistical analysis, injuries primarily characterized by RSP are classified into three groups based on FVC% pred values, while injuries primarily characterized by OSP are classified into three groups based on FEV_1_%pred values. For patients undergoing elective surgery, we perform routine pulmonary function assessments before surgery. The hospitalized patients are all TDL patients who are preparing for surgery, either lobectomy or pneumonectomy. All PFT results are obtained within 24–60 h prior to surgery.

The quality control of PFT: There are standard requirements for the preparation of the site, environment, instruments, materials, subjects, and emergency supplies before conducting the examination. The key to PFT quality lies in quality control, as substandard pulmonary function reports can mislead clinical diagnosis. Daily self-calibration of the pulmonary function instrument is performed, including environmental calibration, which measures temperature, humidity, altitude, and atmospheric pressure in the measurement environment. Before each daily test, a 3 L calibration syringe is used to calibrate the volume of the spirometer to determine the correction factor for volume measurement. A detailed understanding of the subject’s medical history is necessary to determine the indications for PFT and exclude contraindications. The subject should wear loose-fitting clothing to avoid restricting respiratory movements. Reproducibility is an important indicator of well-performed pulmonary function tests. It is required to perform at least three acceptable tests and ensure that the difference between the best and second-best values of FVC and FEV_1_ is less than 0.15 L. During multiple tests, overlapping printing of the F-V curve and T-V curve can be performed.

### Statistical analysis

Perform PFT testing on hospitalized patients diagnosed with TDL. Group patients based on the characteristics of lung function impairment and divide FVC% pred/FEV_1_% pred into three equal segments. Use the Shapiro-Wilk test to determine if the quantitative data is normally distributed. For normally distributed continuous variables, express them as mean ± standard deviation and use T-test. For non-normally distributed continuous variables, use median and interquartile range, and perform Z-test. Present categorical variables as numbers and percentages, analyze them using chi-square (χ2) test or Fisher’s exact test. Analyze the relationship between the declining values of lung function FVC% pred/FEV_1_% pred and the clinical characteristics of TDL patients through testing linear trend. Identify several risk factors closely associated with RSP or OSP based on literature reports, significant differences in univariate analysis, and clinical experience. These factors include gender, age, smoking history, BMI, mMRC, history of tuberculosis, spinal scoliosis, contralateral pulmonary infection, and the median of FVC% pred/FEV_1_% pred three equal segments [1, 9, 10, 12]. Include these factors in a binary multivariate logistic regression analysis to determine their association with the presence of RSP or OSP. Set the significance level at *P*-value < 0.05. Perform all statistical analyses using SPSS 26.0 statistical software package.

## Results

A total of 129 patients were included in the study. Based on the characteristics of lung function, the patients were divided into two groups: RSP group (*n* = 60) and OSP group (*n* = 69), as shown in Fig. 1. Among them, there were 51 males and 78 females, with a mean age of onset of 41.0 ± 14.3 years and a BMI of 21.3 kg/m^2^. Smokers accounted for 14.7% (19 cases), and 5.4% (7 cases) of the patients were alcoholics. The median duration of tuberculosis history in patients was 48.0 months. The main underlying diseases included bronchiectasis (*n* = 3, 21.4%), chronic pulmonary aspergillosis (*n* = 23, 17.8%), multidrug-resistant or extensively drug-resistant (MDR/XDR) tuberculosis (*n* = 18, 14%); diabetes (*n* = 6, 4.7%), chronic liver dysfunction (*n* = 11, 8.5%), and hypertension (*n* = 7, 5.4%). The most common respiratory symptoms were fever (*n* = 15, 11.6%), cough (*n* = 53, 41.1%), massive hemoptysis (*n* = 23, 17.8%), and dyspnea (mMRC ≥ 2, *n* = 34, 26.4%). All patients had one of the following radiographic manifestations: cavitation (*n* = 28, 21.7%), spinal deformity (*n* = 29, 22.5%), contralateral lung infection (*n* = 71, 55%). Compared with patients in the RSP group, patients in the OSP group had a higher BMI (20.3 kg/m^2^ vs. 22.0 kg/m^2^; *P* = 0.003) and longer history of tuberculosis (24 months vs. 84 months; *P* = 0.036), but a smaller proportion of MDR/XDR cases (13% vs. 5%; *P* = 0.023). There were no significant differences in other clinical characteristics, CT manifestations, and laboratory tests between the two groups (*P* > 0.05), Table 1.

**Table 1:** Demographic and clinical characteristics of TDL patients differing in restrictive vs. obstructive ventilatory dysfunction

Variables	Total (*n* = 129, %)	Restrictive spirometric pattern (*n* = 60, %)	Obstructive spirometric pattern (*n* = 69, %)	*P* value
**Sex, male**	51.0(39.5)	24.0(40.0)	27.0(39.1)	0.920
**Age, years**	41.0 ± 14.3	38.5 ± 14.3	43.2 ± 13.9	0.059
**BMI, kg/m2**	21.2 ± 3.2	20.3 ± 3.2	22.0 ± 3.0	0.003
**Smoking history**	19.0(14.7)	10.0(16.7)	9.0(13.0)	0.562
**Alcoholism**	7.0(5.4)	3.0(5.0)	4.0(5.8)	1.000
**Course of tuberculosis, months, months**	48.0(12.0,138)	24.0(12,81)	84.0(12,192)	0.036
**Underlying lung disease**				
Bronchiectasis	3.0(21.4)	2.0(11.8)	3.0(20.0)	0.933
CPA	23.0(17.8)	12.0(20.0)	11.0(15.9)	0.213
MDR/XDR	18.0(14.0)	13.0(21.7)	5.0(7.2)	0.023
**Other comorbidities**				
Diabetes	6.0(4.7)	3.0(5.0)	3.0(4.3)	1.000
Chronic hepatic insufficiency	11.0(8.5)	6.0(10.0)	5.0(7.2)	0.576
Chronic hypertension	7.0(5.4)	4.0(6.7)	3.0(4.3)	0.704
**Chronic pulmonary symptoms**				
Fever	15.0(11.6)	5.0(8.3)	10.0(14.5)	0.276
Sputum	53.0(41.1)	24.0(40.0)	29.0(42.0)	0.815
persistent hemoptysis	23.0(17.8)	9.0(15.0)	14.0(20.3)	0.434
mMRC ≥ 2	34.0(26.4)	11.0(18.3)	23.0(33.3)	0.054
**Chest computed tomographic findings**				
Cavitation	28.0(21.7)	13.0(21.7)	15.0(21.7)	0.992
Spinal state	29.0(22.5)	14.0(23.3)	15.0(21.7)	0.829
Bilateral lung infections	71.0(55)	32.0(53.3)	39.0(56.5)	0.717
**Laboratory findings**				
CRP	9.9(2.4,27.7)	12.7(2.7,33.0)	8.0(1.8,26.0)	0.279
HB	122.3 ± 16.5	125.2 ± 16.8	120.0 ± 15.9	0.069
ALB	38.4 ± 5.3	39.1 ± 5.8	37.7 ± 4.7	0.126

**Abbreviation: BMI: **body mass index;**CPA: **chronic pulmonary aspergillosis; **MDR: **multidrug resistance**; XDR**: extensively-drug resistant; mMRC: **CRP**, C-reaction protein; **HB**: hemoglobin; **ALB**: albumin

The results of PFT showed that in the RSP group, the measured values of FVC and FEV_1_ were (2.2 ± 0.6) L and (1.8 ± 0.5) L, respectively. In the OSP group, the measured values of FVC and FEV_1_ were (2.2 ± 0.6) L and (1.4 ± 0.5) L, respectively. Compared with the RSP group, the measured value of FEV_1_ and FEV_1_%pred were lower in the OSP group, and the between-group comparison was statistically significant (*P* < 0.001), Table 2.

**Table 2:** Values of important indicators in pulmonary function tests

Variables	Total (*n* = 129)	Restrictive spirometric pattern (*n* = 60)	Obstructive spirometric pattern (*n* = 69)	*P* value
**FVC, L**	2.2 ± 0.6	2.2 ± 0.6	2.2 ± 0.6	0.942
**FVC, pred%**	59.8 ± 13.4	57.8 ± 11.7	62.0 ± 16.5	0.1000
**FEV**_**1**_, **L**	1.6(1.2,2.0)	1.8 ± 0.5	1.4 ± 0.5	<0.001
**FEV**_**1**_, **pred%**	52.7 ± 15.4	58.2 ± 12.2	47.8 ± 16.3	<0.001

**Abbreviation: FVC% pred**: forced vital capacity of predicted value of predicted ; **FEV**_**1**_**% pred**: forced expiratory volume in one second of predicted

In patients with RSP, the detection value of FVC% pred is divided into three groups: Group 1 (FVC% pred < 53.0%), Group 2 (53.0% ≤ FVC% pred < 63.1%), and Group 3 (FVC% pred ≥ 63.1%). Among these groups, patients with lower FVC% pred values tend to exhibit higher BMI, a higher proportion of spinal scoliosis, and increased levels of CRP (*P* for trend < 0.001, 0.027, and 0.013, respectively), Table 3.

**Table 3:** RSP patients characteristics by tertile of FVC (% predicted)

FVC (% predicted)	Tertile 1 < 53.0	Tertile 2 53 ≤ - <63.1	Tertile ≥ 63.1	*P* for trend
	(*n* = 21)	(*n* = 20)	(*n* = 19)	
Age, years	41.0 ± 14.8	35.5 ± 12.7	38.9 ± 16.0	0.478
Sex, male	5.0(23.8)	9.0(45.0)	10.0(52.6)	0.152
BMI, kg/m2	21.7 ± 2.4	21.2 ± 3.3	17.9 ± 2.7	<0.001
Smoking history	3.0(14.3)	3.0(15.0)	4.0(21.1)	0.823
Alcoholism	1.0(4.8)	1.0(5.0)	1.0(5.3)	1.000
**Course of tuberculosis, months**,	46.2(39.2,50.5)	58.9(56.2,60.7)	70.7(66.2,74.5)	0.866
**Underlying lung disease**				
Bronchiectasis	1.0(9.5)	0.0(15.0)	1.0 (15.8)	0.803
CPA	3.0 (14.3)	4.0(20.0)	5.0(26.3)	0.637
MDR/XDR	6.0(28.6)	2.0(10.0)	5.0(26.3)	0.307
**Other comorbidities**				
Diabetes	0.0(0)	3.0(15.0)	0.0(0)	0.062
Chronic hepatic insufficiency	4.0(19.0)	1.0(5.0)	1.0(5.3)	0.346
Secondary hypertension	3.0(14.3)	0.0(0)	1.0(5.3)	0.257
**Chronic pulmonary symptoms**				
Fever	1.0(4.8)	1.0(5.0)	3.0(15.8)	0.429
Sputum	10.0(47.6)	4.0(20.0)	10.0(52.6)	0.078
Persistent hemoptysis	2.0(9.5)	4.0(25.0)	3.0(15.8)	0.426
mMRC ≥ 2	4.0(19.0)	2.0(10.0)	5.0(26.3)	0.453
**Chest computed tomographic findings**				
Cavitation	3.0(14.3)	5.0(25.0)	5.0(26.3)	0.630
Spinal state	7.0(36.8)	6.0(30.0)	1.0(4.8)	0.027
Bilateral lung infections	12.0(57.1)	7.0(35.0)	13.0(68.4)	0.102
**Laboratory findings**	9.5(5.9,13.2)	10.1(7.3,15.0)	9.3(5.8,12.6)	0.656
CRP	18.5(3.5,63.2)	16.1(2.7,34.5)	4.5(1.3,14.0)	0.013
HB	125.7 ± 14.2	129.8 ± 14.6	119.7 ± 20.4	0.142
ALB	40.6 ± 6.2	39.4 ± 5.7	37.2 ± 5.2	0.148

**Abbreviation: RSP**: restrictive spirometric pattern; **BMI: **body mass index; **CPA: **chronic Pulmonary Aspergillosis; **MDR: **multidrug Resistance**; XDR**: extensively-drug resistant; mMRC, ModifiedBritish Medical Research Council; **CRP**, C-reaction protein; **HB**: hemoglobin; **ALB**: albumin

The study divided the patients with OSP-related lung damage based on the FEV_1_% pred test values into three tertiles: tertile 1 (FEV_1_% pred < 38.9%), tertile 2 (38.9% ≤FEV_1_% pred<52.0%), and tertile 3 (FEV_1_% pred ≥ 52.0%). Compared to patients in tertiles 2 and 3, those in tertile 1 showed an increasing trend in cough symptoms and contralateral lung infection (*P* for trend = 0.036 and 0.009, respectively), Table 4.

**Table 4:** OSP patients characteristics by tertile of FEV_1_ (% predicted)

FEV_1_ (% predicted)	Tertile 1 < 38.9	Tertile 2 38.9 ≤ - <52.0	Tertile ≥ 52.0	*P* for trend
	(*n* = 23)	(*n* = 23)	(*n* = 23)	
**Age, years**	42.4 ± 13.0	43.6 ± 14.9	43.6 ± 14.4	0.944
**Sex, male**	13.0(56.5)	7.0(30.4)	7.0(30.4)	0.112
**BMI, kg/m2**	20.9 ± 2.3	22.6 ± 3.5	22.5 ± 2.9	0.122
**Smoking history**	5.0(21.7)	3.0(13.0)	1.0(4.3)	0.272
**Alcoholism**	0.0(0)	3.0(13.0)	1.0(4.3)	0.313
**Course of tuberculosis, months, months**	96.0(12.0,156)	48.0(12.0,192)	108(36.0,240)	0.594
**Underlying lung disease**				
Bronchiectasis	1.0 (4.3)	1.0(4.3)	1.0(4.3)	1.000
CPA	4.0 (17.4)	4.0(17.4)	3.0(13.0)	1.000
MDR/XDR	1.0(4.3)	2.0(8.7)	2.0(8.7)	1.000
**Other comorbidities**				
Diabetes	0.0(0)	2.0(8.7)	1.0(4.3)	0.768
Chronic hepatic insufficiency	2.0(8.7)	2.0(8.7)	1.0(4.3)	1.000
Secondary hypertension	0(0)	2(8.7)	1(4.3)	0.768
**Chronic pulmonary symptoms**				
Fever	3.0(13.0)	2.0(8.7)	5.0(21.7)	0.580
Sputum	8.0(34.8)	9.0(39.1)	16.0(69.6)	0.036
Persistent hemoptysis	5.0(21.7)	4.0(17.4)	5.0(21.7)	1.000
mMRC ≥ 2	8.0(34.8)	6.0(26.1)	9.0(39.1)	0.633
**Chest computed tomographic findings**				
Cavitation	4.0(17.4)	6.0(26.1)	5.0(21.7)	0.933
Spinal state	10(43.5)	1(4.3)	4(17.4)	0.006
Bilateral lung infections	9.0(39.1)	15(65.2)	19(82.6)	0.009
**Laboratory findings**				
CRP	15.8(7.3,39.3)	6.8(2.7,29.5)	9.1(0.5,16.6)	0.470
HB	121.9 ± 16.3	117.5 ± 13.5	123.2 ± 19.8	0.838
ALB	37.2 ± 4.9	36.9 ± 5.2	38.1 ± 4.8	0.604

**Abbreviation: OSP**: obstructive spirometric pattern; **BMI: **body mass index;**CPA: **chronic pulmonary aspergillosis; **MDR: **multidrug Resistance**; XDR**: extensively-drug resistant; mMRC, ModifiedBritish Medical Research Council; **CRP**, C-reaction protein; **HB**: hemoglobin; **ALB**: album

Binary logistic regression analysis demonstrated that a higher FEV_1_% pred was associated with a lower odds ratio (aOR 0.060, 95% CI 0.018–0.204, *P*<0.001) for OSP patients. Trend toward a lower OR for patients in the OSP group with increasing FEV_1_% pred, Table 5.

**Table 5:** Trends between restrictive and obstructive ventilatory dysfunction and values of FVC and FEV_1_ tests

Variables	Q_1_	Q_2_	Q_3_	*P* for trend
**FVC% pred (RSP 0 /1)**	1.000	2.834(1.004–7.995)	1.504(0.523–4.320)	0.505
**FEV**_**1**_ **% pred** **(OSP 0 /1)**	1.000	0.073(0.022–0.248)	0.060(0.018–0.204)	<0.001

Note: The independent variables in the binary regression model include: age, gender, BMI, smoking history, mMRC, previous history of pulmonary tuberculosis, spinal state, bilateral lung infections

## Discussion

TDL is the terminal stage of progression in tuberculosis patients, with a high clinical mortality rate [8, 21, 22]. Respiratory physicians often use PFT testing to predict the condition and progression of chronic lung diseases. In TDL patients, we observed that Patients with high BMI, a higher proportion of spinal scoliosis, and abnormal elevation of CRP levels were more likely to have reduced FVC. Patients with a decrease in FEV_1_% pred also exhibit more frequent cough symptoms and a higher proportion of contralateral lung infections.

Furthermore, we observed that TDL patients with RSP predominantly have a higher proportion of combined MDR/XDR than OSP predominantly. This suggests that MDR/XDR may accelerate the progression of pulmonary damage, leading to a more restrictive pattern. However, patients with OSP group have significantly longer histories of tuberculosis compared with RSP group. This may be due to long-term invasion of lung tissue by *Mycobacterium tuberculosis*, leading to alveolar collapse, type II epithelial cell proliferation, collagen fiber hyperplasia in the alveolar walls and pulmonary interstitium, resulting in shrinkage and fibrosis of lung tissue, ultimately leading to loss of pulmonary function.

In our study, we observed a negative correlation between FVC% pred and BMI in patients with RSP group. Studies have shown that the higher the BMI, the lower the FVC, which is consistent with our findings [23, 24]. We also found a higher proportion of spinal curvature in CT imaging. Spinal curvature leads to thoracic deformity, long-term restriction of lung tissue and thoracic expansion, resulting in restrictive ventilation impairment. In addition, as FVC% pred decreased, CRP levels gradually returned to normal range from abnormal elevation. CRP level is less influenced by factors such as age, immune status, and medications. It rapidly decreases and returns to normal only when infections are controlled, making it a reliable and sensitive marker of inflammatory response. In TDL patients, pulmonary inflammation initially leads to immune imbalance, with immune cells accumulating in the lung interstitium, causing damage to epithelial and endothelial cells. Subsequently, abnormal immune repair induces and activates epithelial-mesenchymal transition, triggering fibrotic reaction, resulting in progressive and irreversible disruption of lung structure and restricted ventilation of alveolar tissue, manifested as a decrease in FVC% pred. In conclusion, we observed a possible negative correlation between FVC% pred and BMI.

This study also divided the measurement of FEV_1_% pred in OSP patients into three groups. FEV_1_% pred is a representative indicator of obstructive pulmonary function impairment. With a decrease in FEV_1_% pred measurement, patients present more frequent cough symptoms. Coughing is a defensive neural reflex of the body, which helps to clear respiratory secretions and harmful substances. Cough in patients is often caused by increased airway secretions. The lower the proportion of contralateral lung infection on CT, the higher the measured value of FEV_1_% pred. These findings may suggest a relationship between FEV_1_% pred measurement and increased airway secretions caused by lung infection.

In previous studies, the lung function measurements of TDL were based on individual indicators such as FVC and FEV_1_ [2, 25, 26]. There was no discussion on classification based on the pattern of lung function impairment. Our study indicates that classifying TDL disease progression based on the characteristics and severity of lung function impairment may be a feasible alternative method.

The current study has certain limitations. Firstly, this study is a retrospective observational study conducted in a single center, which may lead to selection bias. Secondly, not all patients underwent PFT screening, mainly due to the low attention of tuberculosis specialists in our hospital to PFT in TDL patients, and the possibility of increased nosocomial infection due to the contagiousness of pulmonary tuberculosis. Therefore, some patients did not undergo PFT. Thirdly, when patients experienced massive hemoptysis, immediate surgery was often performed without PFT, and this is also a contraindication for PFT.

## Conclusion

Clinical characteristics of TDL patients can be differentiated based on different features of lung ventilation injury. Patients with high BMI, a higher proportion of spinal scoliosis, and abnormal elevation of CRP levels were more likely to have reduced FVC. In addition, patients with a decrease in FEV_1_% pred have more frequent cough symptoms and a higher proportion of contralateral lung infection. The higher the FEV_1_% pred in pulmonary function testing, the lower the likelihood of OSP occurrence. To predict the condition and progression of this disease based on the characteristics of lung function impairment and clinical manifestations in TDL patients, further large-scale clinical practice is needed for validation.

## Data Availability

The data that support the findings of this study are not openly available due to reasons of sensitivity and are available from the corresponding author upon reasonable request.

## Abbreviations

TDL: Tuberculosis destroyed lung
CPA: chronic pulmonary aspergillosis
OR: odds ratio
CI: confidence interval
TB: tuberculosis
CT: computerized tomography
CRP: C-reaction protein
mMRC: Modified British Medical Research Council
MDR-TB: multidrug-resistant tuberculosis
XDR-TB: extensively drug-resistant tuberculosis
BMI: body mass index
FEV_1_% pred: forced expiratory volume in one second of predicted
FVC% pred: forced vital capacity of predicted
